# Coma and cerebral imaging

**DOI:** 10.1186/s40064-015-0869-y

**Published:** 2015-04-16

**Authors:** Walter F Haupt, Hans Christian Hansen, Rudolf W C Janzen, Raimund Firsching, Norbert Galldiks

**Affiliations:** Department of Neurology, University of Cologne, Kerpener St. 62, 50937 Cologne, Germany; Department of Neurology and Psychiatry, Friedrich-Ebert Krankenhaus, Friesenstr. 11, 24534 Neumünster, Germany; Landwehrweg 12c, 62350 Bad Homburg, Germany; Department of Neurosurgery, Otto-von-Guericke University Magdeburg, Leipzigerstr 44, 39120 Magdeburg, Germany; Research Center Juelich, 52425 Juelich, Germany

**Keywords:** Coma, Brain disease, Brain death, Computed tomography (CT), Magnetic Resonance Imaging (MRI), Functional Magnetic Resonance Imaging (fMRI)

## Abstract

The clinical sign of coma is a common feature in critical care medicine. However, little information has been put forth on the correlations between coma and cerebral imaging methods. The purpose of the article is to compile the available information derived from various imaging methods and placing it in a context of clinical knowledge of coma and related states. The definition of coma and the cerebral structures responsible for consciousness are described; the mechanisms of cerebral lesions leading to impaired consciousness and coma are explained. Cerebral imaging methods provide a large array of information on the structural changes of brain tissue in the various diseases leading to coma. Circumscript lesions produce space-occupying masses that displace the brain, ultimately leading to various types of herniation. Generalized disease of the brain usually leads to diffuse brain swelling which also can cause herniation. Epileptic states, however, rarely are detectable by imaging methods and mandate EEG examinations. Another important aspect of imaging in coma is the increasing use of functional imaging methods, which can detect the function of loss of function in various areas of the brain and render information on the extent and severity of brain damage as well as on the prognosis of disease. The MRI methods of ^1^H-spectroscopy and diffusion tensor imaging may provide more functional information in the future.

## Introduction

Coma is one of the most common clinical signs in critical care medicine and is observed in all areas of critical care. Unfortunately, little interest seems to be directed towards this ubiquitous clinical sign, and few intensivists take the time to closely observe and document phenomena of impaired consciousness and come in their patients. Usually, only global scores of impaired consciousness are documented. This is somewhat astonishing considering the fact that the functional impairment of brain function in any disease course probably has the greatest impact on the long-term outcome of the individual patient.

The rapid development of cerebral imaging methods in medicine in general and intensive care medicine in particular has vastly improved our understanding of cerebral lesions and their development in the disease course. On the other hand, only little attention has been directed toward the information on consciousness and come that can be derived from imaging methods.

While cerebral imaging is able to detect and assess the localization and size of space-occupying lesions, it can render only little information on the functional impairment of the brain. These functional deficits are usually better studied by neurophysiological methods, such as EEG and evoked potentials. These methods are far better suited to make statements as to the functional disturbance and its prognosis.

The authors have attempted to compile an overview of the contribution of brain imaging methods to the knowledge of the phenomenon “coma”. This collection of data can only shed a spotlight on various areas of scientific investigation and cannot provide an exhaustive lexical database on brain imaging and coma. Keeping these limitations in mind, the collection may increase the interest in the contribution of cerebral imaging methods in the assessment of coma.

### Causes of coma: etiologic factors

Coma is defined as a severe disturbance of consciousness, which precludes awakening, and the directed movement of extremities. The comatose person demonstrates closed eyes and shows no purposeful reaction to noxious stimuli. The quantitative reduction of wakefulness, or better of arousal function, is the main feature of this condition. Many other qualitative disturbances of consciousness may occur before the manifestation of the state of coma. If both phenomena appear together in a fluctuating or alternating manner, the diagnosis of an organic psychosis of the delirium type should be considered. The broad spectrum of etiologies overlaps and is not specific for either qualitative or quantitative disturbances.

In older age, typical etiological factors include metabolic disturbances such as hypoglycemia or hyperglycemia, electrolyte imbalance as well as single and multiple organ failure including hepatic or renal failure and finally focal and global cerebral perfusion deficits especially in stroke or anoxia following resuscitation (Cloyd et al. 2006). In younger patients, traumatic brain injuries and intoxications due to alcohol, drugs or medications are more prevalent.

Other metabolic disorders with secondary effects on the CNS should always be considered. These are not always immediately apparent in laboratory examinations and are collectively designated as encephalopathy.

### Pathophysiology of coma

While no circumscript brain area is responsible for consciousness, the neurotopical localization can be found in the ascending reticular activating system (ARAS). Whenever this system is functionally impaired bilaterally, one must anticipate disturbances of consciousness ultimately attaining the degree of coma. The ARAS connects the thalamic and subthalamic nuclei with the reticular intermediary grey substance of the spinal cord. Here, the etiology and exact localization of the functional neuronal disturbance in the ARAS are not especially important: reversible metabolic CNS disease in the context of metabolic derangement is just as well possible as structural lesions along the thalamic loop structures.

**Figure 1:**
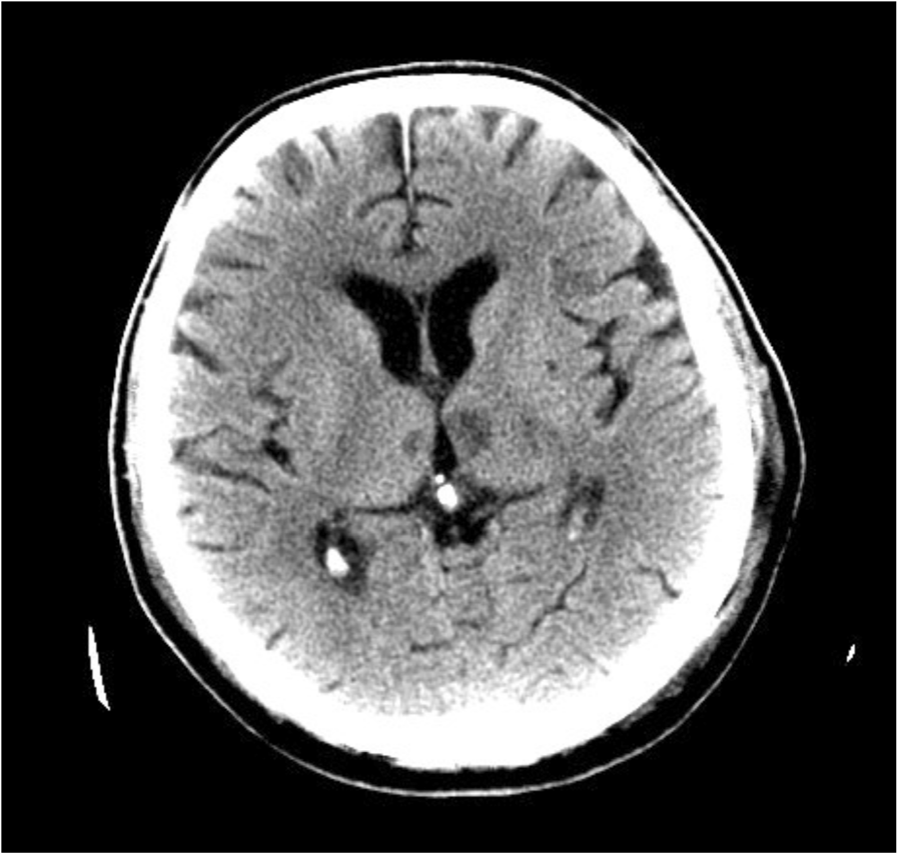
68 year- old patient with bilateral embolic thalamic infarctions causing coma.

However, infratentorial space-occupying lesions with consecutive brain stem compression cause disturbances of consciousness through secondary functional disturbances within the ARAS (e.g., cerebellar hemorrhage). Even very small lesions, strategically placed bilaterally in the ARAS, can produce coma, e.g. in cases of small bilateral infarctions in the thalamo-mesencephalic border region or in the thalamic nuclei (Figure 1). As a rule, circumscript brain stem lesions that do not reach the midline structures, do not lead to quantitative disturbances of consciousness to the extent of coma, therefore, other etiologies should be considered.

**Figure 2:**
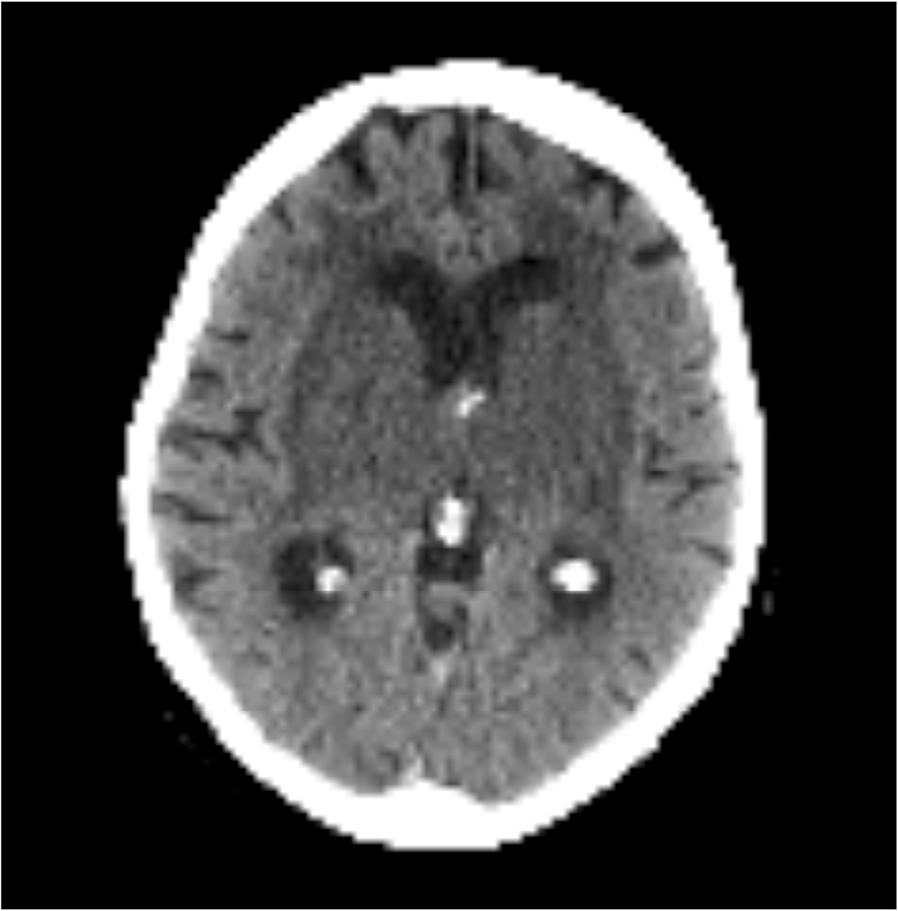
90 year-old patient with thrombosis of inner veins, causing bilateral thalamic and basal ganglia edema, resulting in coma.

Unilateral hemispheric lesions do not produce coma as long as they do not compromise the midline structures or lead to a secondary impairment of the brainstem structures. Both features can be caused by progressive focal cerebral edema and spreading of a lesion to the contralateral side (Figure 2). This progressive functional impairment of brain tissue may cause lesions that are below the resolution of neuroimaging methods and therefore can remain undetected in the initial phase of disease.

Secondary brain lesions are caused by displacement of brain tissue: The space- occupying lesions lead to lateral displacement (lateral herniation) and downward directed herniation toward the brainstem (uncal herniation), and the immediately life-threatening lower herniation of the cerebellar tonsils which often occurs in the preterminal phase of disease. The latter compromises the lower brain stem nuclei, which are essential for the regulation of respiratory function and blood pressure regulation.

Another important event immediately preceding death in coma is the development of venous congestive swelling and hemorrhages (Duret’s hemorrhages) due to impairment of drainage though swelling of the brainstem tissue. The degree of displacement of the midline (in mm, measured by CCT) has been correlated in vivo with the depth of coma.

In the detection of primary cerebral lesions, the terms of focal and lateralizing signs have been introduced. Usually, ipsilateral pupillary enlargement occurs in uncal herniation, due to the compression of the ipsilateral oculomotor nerve. The rare appearance of contralateral mydriasis is explained by the fact that some space occupying lesions develop less downward, but rather predominantly lateral displacement, resulting in a tipping of the neuraxis. Instead of an obstruction of the ipsilateral parts of the perimesencephalic cisterns, the contralateral fluid compartments are compressed which can be followed in imaging tests. Thus, “false localizing signs” such as a dilated contralateral pupil or spastic signs ipsilateral to the primary cerebral lesion can be well explained and corroborated by neuroradiological findings. In neuropathological terms, this corresponds to the pressure of the brainstem on the tentorium (Kernohan’s notch), and clinically, this is probably closely correlated to the appearance of extensor spasms and posturing.

### Clinical assessment and pitfalls

The initial assessment of the clinical presentation is dominated by the neurological and general clinical examination. Aside from determining the vital signs (e.g., breathing, circulation, temperature), the presence of neck stiffness and focal neurological deficits must be recorded. The Tables 1 and 2 give an overview of the clinical examination and its results.

**Table 1:** **Initial clinical assessment (Pseudocoma is not included since it can mimic any type of coma)**

**Clinical impression**	**Etiology**
Coma without focal signs or neck stiffness	anoxic-ischemic
	toxic-.metabolic
	(post-) epileptic
	inflammatory
	hypothermia
	traumatic brain injury
Coma with neck stiffness	subarachnoid hemorrhage
	meningitis
	meningoencephalitis
Coma with focal signs	neurovascular (arterial infarctions, hemorrhages, venous thrombosis) abscess, tumor, traumatic brain injury

**Table 2:** **Correlation of localization and clinical signs in comatose states**

**Localization of lesion leading to coma**	**Clinical findings**
Coma with bilateral hemispheric lesions	Quadriparesis with symmetrical alteration of muscle tone and reflexes (extensor and flexor posturing, Babinski’s sign)
	Seizures, myoclonus possible
	Brain stem and pupillary reflexes are intact
Coma with unilateral supratentorial lesion and secondary brain stem lesion	Ipsilateral oculomotor nerve palsy and contralateral hemiparesis evolving to quadriparesis
Coma with primary brain stem lesion	Quadriparesis with an asymmetrical muscle tone und reflex abnormalities (extensor and flexor posturing Babinski’s sign)
	Pathological brain stem reflexes
Coma due to toxic and metabolic disease	Tetraparesis with symmetrical muscle tone and reflex abnormalities (extensor and flexor posturing Babinski’s sign)
	Pathological brain stem reflexes and pupillary reflexes intact (except in opiate or sympathicomimetic intoxications)
	Myoclonus or seizures possible

Psychogenic disturbances of consciousness are usually dissociative in nature and only rarely factitious disease. They can mimic any coma-like situation and often also present with focal neurological signs such as speech arrest or sensory-motor hemisyndromes. The verification of the psychogenic nature of these signs is very difficult to achieve, and often evolves over time, after all neurophysiological tests have failed to detect any functional disturbance. Imaging tests invariably demonstrate normal findings or may detect older premorbid conditions that are irrelevant to the presented condition of disturbed consciousness. These patients often demonstrate marked fluctuations of the neurological findings, especially dependent on the attention of the examiner, and often one finds a psychiatric history or a special emotional burden. Reactivation of a traumatic experience in the context of a posttraumatic stress disorder (PTSD) is a common cause for these dissociative states.

**Figure 3:**
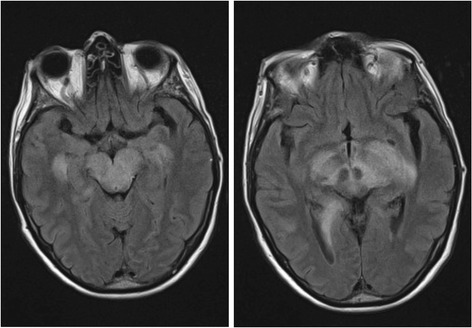
45 year-old patient with coma and lymphocytic pleocytosis in CSF, caused by immune-mediated encephalitis.

Even if neurological focal signs are completely absent, multilocular cerebral lesions remain a possible differential diagnosis and must be sought using imaging methods (e.g., venous thrombosis with congestive hemorrhages and infarctions, bilateral subdural hemorrhages, bacterial meningitis with infarctions, sepsis with focal encephalitis, or immune-mediated encephalitis) (Figure 3).

Furthermore, disorders of consciousness are frequently seen as secondary epileptic complications such as generalized epileptic seizures after stroke. The recovery phase is usually associated with recovery from impaired consciousness during the next few hours, however, in elderly and multimorbid patients, protracted recovery can occur over several days (Cloyd et al. 2006). These states must be distinguished from persisting epileptic states, which require urgent treatment for non-convulsive status epilepticus. This non-convulsive status can be diagnosed by EEG only and cannot be detected by imaging methods.

### Epileptic phenomena in the context of imaging and coma

Coma-like situations regularly occur in the context of epileptic seizures during the postictal phase (see below). As far as danger to life is concerned, the generalized and persistent epileptic functional disturbances are most important. These functional disturbances are accompanied by neuronal necrosis and later by life-threatening brain swelling in combination with cerebral lactacidosis, which cannot be measured by clinical methods. Even regularly reoccurring singular epileptic seizures can produce relevant temporal lobe lesions (hippocampal sclerosis), which are occasionally detectable by imaging methods (Duncan 2002).

Although the metabolic exhaustion can be detected in the form of tissue diffusion abnormalities (cytotoxic edema) in severe cases, imaging methods at present usually do not provide relevant information for the diagnosis of epileptic functional disturbances (Wieshmann et al. 1997). The “gold standard” for the detection of epileptic phenomena remains the EEG, which can verify the diagnosis quickly, determines the adequate therapy, and monitors the effect and success of treatment. However, focal epileptic phenomena, even at the intensity of an overt status epilepticus (simple or partial) in rare cases can go undetected in the EEG due to electrode or focus geometry.

Imaging procedures are necessary in the diagnostic evaluation patients with epileptic states to detect or rule out focal cerebral lesions, which might cause increased focal seizure susceptibility. They are irreplaceable to rule out structural cerebral lesions in the case of de novo onset epileptic seizures. This is especially important in prolonged or persistent coma and in the differential diagnosis of status epilepticus. In all cases of generalized epileptic seizures, the ensuing “postictal” clouding of consciousness must be differentiated, which remits continuously and leads to complete remission within 15 to 30 minutes in uncomplicated cases. In rare cases, especially in elderly patients, the clouding of consciousness can persist for several days before it finally remits.

Psychogenic, so-called dissociative impairment of consciousness, at first sight, often imitates epileptic or post-epileptic states. Quite often, this diagnosis can be found in patients with epileptic seizures as co-morbidity, and regularly leads to recurring coma-like episodes. Here, cerebral imaging tests are necessary to rule out an “organic cause”, even though the diagnosis is secured by clinical examination.

### Functional imaging in persistent vegetative state and minimally conscious state

Since the advent of the CT and MRI, cerebral imaging methods were utilized to visualize the structure of the brain. The more recent developments now make it possible to also visualize the function of the brain, thus bridging the gap between structure and function. In the case of severe brain damage leading to protracted coma in the form of persistent vegetative state (PVS) or the minimally conscious state (MCS), functional imaging methods have provided a host of new insights into these syndromes.

#### PET and SPECT

Imaging methods have been used for over 20 years to detect the extent of cerebral damage in severe diseases of the brain. The method of Positron-Emission-Tomography (PET), most frequently using the tracer ^18^ F-2-Fluoro-2-deoxy-D-glucose (FDG), as well as SPECT (single photon emission tomography) has been used for these purposes. The methods provide information on regional and global function of the brain. In coma and related conditions, they usually show severe global decrease of brain activity.

On examination of patients in PVS using FDG PET, which measures the cerebral glucose metabolism, the global cerebral glucose metabolism was reduced by approximately 50% (Schiff et al. 2002). In addition to this global reduction of glucose metabolism, the investigators also found distinctive regional metabolic patterns with marked hypometabolism. An especially low metabolic rate was found bilaterally in the frontal and temporoparietal cortex (Laureys et al. 2002). In a further study using FDG PET and SPECT, it was found that PVS patients demonstrated only minor hypometabolic alterations in the cerebellar vermis when compared to normal controls. This finding demonstrates that the infratentorial region of the brain is relatively resistant to the effects of severe brain damage.

The radiopharmacon ^11^C-flumazenil can be used in PET as a marker to examine the functional integrity of the brain. A patient suffering from PVS demonstrated marked bilaterally symmetrical loss of supratentorial cortical neurons when examined with this tracer (Rudolf et al. 2000). The metabolic pattern of infratentorial structures and the cerebellum, however, was similar to that of normal persons.

Imaging methods have also been implemented to distinguish between coma-like states such as PVS and MCS. In patients in MCS, the global and regional metabolic rates were not significantly different from those in PVS. Therefore, the differentiation between PVS and MCS by comparison of metabolic rates alone, is highly problematic (Coleman et al. 2005).

#### Functional MRI

In cases where it is impossible for the patient to produce a motor response to exteroceptive stimuli, it is equally impossible to detect a response to outside stimuli by clinical examination. Functional MRI (fMRI) has been implemented to overcome this dilemma. The goal was to measure a change in brain activity in response to stimuli even if a motor response of the patient was not possible. FMRI is a method to examine activated brain areas with high spatial resolution. The method is used for a number of different motor, sensory, and neuropsychological functions. The activation of a certain brain area, which is activated during a specific task, leads to a change in blood oxygenation. This, on the other hand, leads to a change in the magnetic properties of the hemoglobin molecules, which can be detected by MRI. The signal, which is dependent on the oxygen level of the blood, is called the BOLD (blood oxygen level dependent) signal. In this context, the examination is based on the observation that pure thought (imagination) of motor functions leads to activation of certain cerebral areas. The increased oxygen consumption leads to a change of the BOLD signal, which is detected by MRI.

The regions activated by thought tasks could be compared between healthy subjects and patients suffering from PVS or MCS. Owen et al. (Owen et al. 2006) and Laureys et al. (Laureys et al. 2002) as well as other investigators were able to demonstrate reactions of the brain that were similar to those of healthy persons who are only imagining a motor task. By this method it becomes possible to detect cerebral responses to outside stimuli in patients who are unable to perform any kind of motor function, neither movement nor speech.

In an investigation by Owen et al. (Owen et al. 2006) in a patient fulfilling the criteria of PVS, spoken sentences were first offered which lead to activation of speech centers as can be seen in normal subjects. This activation of task-specific brain regions, however, was not considered to be a sufficiently reliable sign of preserved consciousness, because some investigations have shown that certain functions of speech perception are still preserved in comatose states. In a further experiment, the patient was asked to imaging playing tennis, then to imagine walking through her home. The regional activation of brain areas in the two tasks was identical to the activation patterns in healthy subjects. The authors concluded that the patient must be able to understand the commands and imagine different motor tasks. It became clear that patients were able to willfully modulate their brain function. This ability requires a conscious perception of the surroundings and therefore proves consciousness.

Functional MRI makes it possible to visualize consciously imagined motor tasks, which are initiated by commands from outside without using any kind of motor function. It becomes possible to detect consciousness without utilizing any motor function.

### Imaging in traumatic brain injury

Since the discovery of x-rays more than 100 years ago, and after a rapid development of neuroradiological techniques, we now possess a number of methods, which have proven useful in the treatment of traumatic brain injury (TBI). The most important methods are native x-ray diagnostics, angiography, computed tomography (CT), and magnetic resonance imaging (MRI).

The Ethics Committee of the University of Magdeburg approved the anonymous evaluation of data of comatose patients after head injury for scientific purposes without consent of the patients or their legal guardians (decision 131 of 1997).

The indications and relevance of these four examination methods will be discussed in the order of their historical development.

#### Skull x-ray

Today, the skull x-ray only plays a historical role in the treatment of TBI patients. In patients with impaired consciousness and a skull fracture, the probability of an intracranial hemorrhage amounts to 1 in 4 (Mendelow et al. 1983). On the other hand, patients with clear consciousness and no skull fracture only show a probability of 1:6000 of developing an intracranial hemorrhage. If a skull fracture is detected by skull x-ray in a patient with clear consciousness, he should be transferred to an institution where a CT scan can be performed soon (Brandt et al. 2009). The fractures detectable by skull x-ray are divided into linear fractures, impression fractures (or expression fractures) and burst fractures. The location of the fracture may be classified as fractures of the convexity, fractures of the skull base, frontobasal fractures, temporal bone fractures, and fractures of the condyles.

#### Angiography

Since the advent of CT and MRI, angiographies are performed only rarely in TBI. CT angiography and MRI angiography have such high resolution so that even minor vascular injuries can be detected. The indications for conventional angiography may be summarized as follows: suspicion of a vascular lesion, traumatic vascular occlusion, traumatic aneurysm, vascular dissection, carotid sinus fistula, and exclusion of an aneurysm in subarachnoid hemorrhage (which may have precipitated the TBI).

#### Computed tomography

To date, the CT scan is the method of choice in TBI and cases of unconsciousness since it can detect density changes between brain tissue and acute hemorrhages with high resolution (Brandt et al. 2009). A CT scan should be performed as soon as possible following TBI. Vital functions, breathing and circulation must be secured prior to any imaging. Since every unconscious patient should be intubated to prevent aspiration, the CT scan must be performed under mechanical respiration. Indications for CT scans following TBI are coma, impaired consciousness, other neurological abnormalities, epileptic seizures, skull fracture confirmed in the skull x-ray, suspicion of impression fracture or penetrating injury, suspicion of cerebrospinal fluid fistula, differential diagnosis, questionable cases, follow-up scanning during the clinical course.

The urgency for CT scanning is governed by the dynamic evolution of intracranial hemorrhage. Extradural hematomas require more than one hour to become space occupying. However, in the second hour they become space occupying and mortality increases with the increasing time interval between TBI and CT scan. Space-occupying subdural hemorrhage can appear anytime during the clinical course and can reach substantial size. The earlier they appear, the accompanying collateral damage to the brain and mortality increases. Since space-occupying subdural hematomas, which appear during the first hour, have a mortality of 88% despite immediate operative treatment, only few patients profit from early diagnosis. From these observations it follows that a CT scan should be performed within the first hour after trauma. Since comatose patients cannot report additional injuries, multiple injuries must always be suspected. Bleeding into the major body cavities, thorax and abdomen should be identified or ruled out immediately. Therefore, comatose patients should always be admitted only to hospitals that offer competent neurosurgical care of intracranial injuries as well as care to injuries to abdomen, thorax, facial skull, and extremities.

Aside from the detection of intracranial injuries, especially hemorrhages, the CT can easily detect frontobasal injuries with clinically relevant CSF leakage. Petrous bone fractures with otogenic CSF leakage usually resolve within 3 weeks with rare exceptions (Cooper 1982) whereas frontobasal CSF leaks require operative closure as they can lead to life-threatening meningitis even years later. Occasionally, the detection of frontobasal leaks in the frontobasal region can be difficult. The CT offers the capability to perform cisternography with injection of contrast media to the subarachnoid cavity and demonstration of the CSF leak.

#### Specific CT findings - epidural hematoma

A biconvex hemorrhage, developing at the inside of the skull wall, is typical for an epidural hematoma. Occasionally, epidural hematomas require some time to develop which may render the initial CT scan normal and can only be detected on repeat exam. In cases of persisting coma and neurological deterioration, a follow-up CT scan should be performed. Since this deterioration usually occurs within a period of 12 hours (Frowein 1991), the follow-up CT scan should be performed within this time span.

#### Specific CT findings - subdural hematoma

The development time of these hematomas is especially variable. They can be detected immediately following trauma, but can also appear months later. With the assistance of histological investigations, the following classification has proven to be valuable (Firsching et al. 1989): i) acute subdural hematomas within the first 24 h after trauma; ii) subacute subdural hematomas between 2 and 10 days following TBI; and iii) chronic subdural hematoma after the 10^th^ day following TBI. Depending on the time interval, the density of the hematomas is variable. Acute hemorrhage occurs usually hyperintense on CT scan, whereas chronic hemorrhage can appear isodense and, therefore, difficult to detect.

#### Specific CT findings - intracerebral hemorrhage

Hemorrhages within the brain tissue can appear in any region of the brain. In contrast to earlier opinion, they can also occur in the brain stem (Firsching et al. 1998). They can appear as single or multiple lesions of variable size. In 25% of the cases with first identification of a contusion, a follow-up CT scan can detect an increase in size (Frowein 1991). The size of the contusion does not necessarily correlate with mortality. Whereas the majority of contusions increase in size over the first 12 hours (Frowein 1991), one can also find intracerebral posttraumatic bleeds, which appear after days or weeks, known as posttraumatic late apoplexy (Bollinger 1891). CT scans are most important for the diagnosis of brain death since the prerequisite for the diagnosis of brain death is the demonstration of a primary cerebral lesion. In rare cases a primary infratentorial brain lesion may lead to the clinical signs of brain death, i.e., coma and apnoeic loss of cranial nerve function as signs of the loss of brain stem function but simultaneously preserved supratentorial brain function as eventually demonstrated by a preserved EEG. In most countries of the world the diagnosis of brain death requires evidence of the death of the entire brain and not only of the brain stem. These rare cases can only be recognized when early CT identifies a primary infratentorial lesion.

#### Specific CT findings - diffuse brain damage

The term “diffuse axonal lesion” following TBI as a CT scan finding is used in a confusing way in the literature. Strich et al. first described diffuse axonal lesions histologically in 1956 (Cooper 1982). Diffuse brain injuries were a major cause of coma and death. Under this tenet, Gennarelli recommended to assume a diffuse brain lesion when the patient remained in coma for more than 6 hours and the CT scan did not demonstrate a space-occupying lesion (Cooper 1982). Following this idea, Marshall et al. recommended a scale of CT findings of diffuse brain injury in which the severity of signs of pressure was differentiated by determination of the degree of midline shift and compression of the perimesencephalic cisterns (Marshall et al. 1991). He distinguished 4 grades of CT findings; grade 4 indicated an intracranial hemorrhage of at least 25 cm^2^. While a prognostic relevance of the classification has been claimed, its practical value is limited. An extradural hematoma of more than 25 cm^2^ may be fatal when removed too late and may be survived without consequence when removed in time. Due to artifacts caused by the petrous bone structures lesions in the posterior fossa are not adequately depicted by CT.

#### Magnetic resonance imaging

**Figure 4:**
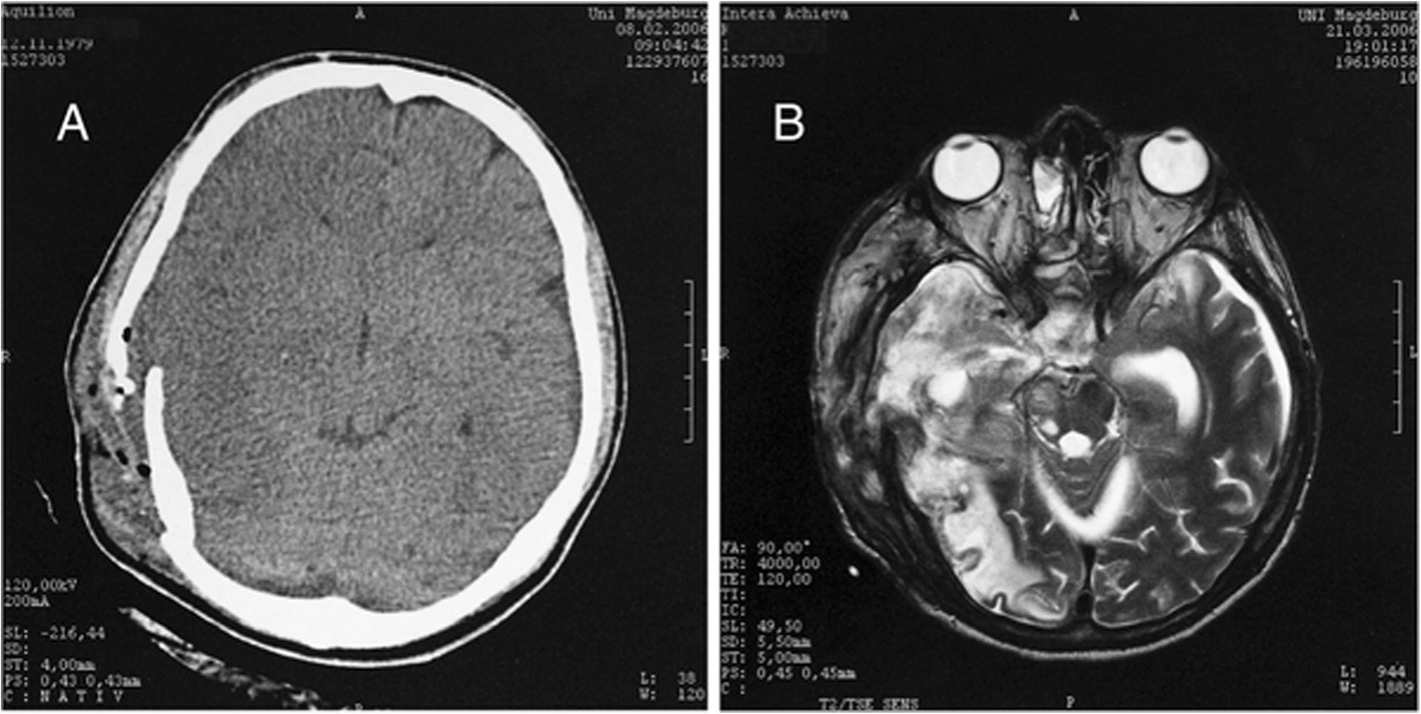
26 y/o patient with traumatic brain injury. **A**. CT scan only shows impression fracture and mild swelling. **B**. MRI shows severe contusion of right temporal lobe and bilateral mesencephalic lesions causing coma.

**Figure 5:**
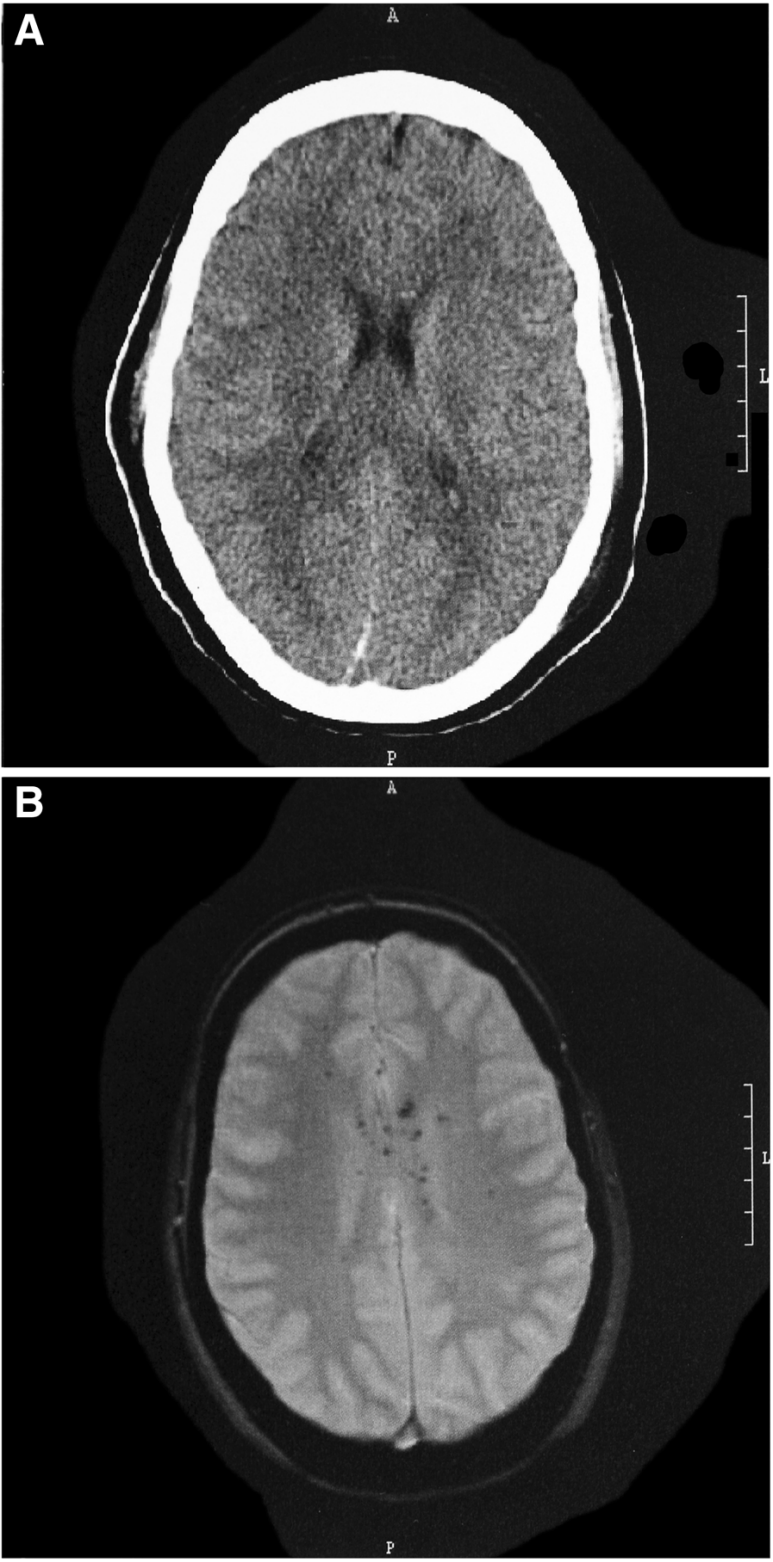
16 y/o patient with traumatic brain injury. **A**. CT shows no lesion. **B**. MRI shows multiple disseminated microbleeds in the T2* sequences.

MRI is not the diagnostic method of first choice in emergencies, as it requires significantly longer scanning times than CT scans. Also, fractures cannot be depicted as well as with CT scanning. The advantage of MRI is the superior imaging of soft tissue and the more precise demonstration of lesions of the posterior fossa (Figure 4A and B). Severe lesions of the brain stem, which cannot be found using CT, can be identified by MRI (Figure 5A and B). Since these lesions are of great importance for prognosis, the advantage of MRI lies in its superior prognostic value. MRI of comatose patients requires antimagnetic equipment, e.g., respirator, intravenous pumps, to maintain vital functions. Other indications for MRI are the high-resolution imaging of CSF using CISS sequences for the detection of CSF fistulas. The T2* sequences are of special value as they can detect even smallest traces of hemorrhages even after many years.

#### Prognostic value of MRI

Several large prospective MRI studies in comatose patients after TBI revealed the exceptional prognostic value of MRI (Firsching et al. 1998, 2001; Luchtmann et al. 2013). These studies led to a statistically significant scale of MRI findings, which correlated with mortality and quality of life. The following grades were defined: grade I, supratentorial lesion only, no brain stem lesion; grade II, unilateral brain stem lesion in variable location with and without additional grade I lesion; grade III, bilateral mesencephalic lesion with or without additional grade II lesion; and grade IV, bilateral pontine lesion with or without additional grade III lesion. Patients who remained in coma for 24 hours after trauma with the minimal sedation needed to ensure adequate respiration had a probability of 60% of a brain stem lesion that had not been detected by CT scan.

### Terminal coma and complete irreversible loss of all brain functions (“brain death”)

The examination of the course of coma states and the prognostication of comatose patients was performed since the 1950s by neurological examination, EEG testing and later by evoked potential testing. Additionally monitoring of the intracranial pressure and/or metabolic measurements has been performed. Imaging of the brain was restricted to the visualization of indirect signs derived from the examinations of intra- and extracranial blood vessels by angiography. Cerebral perfusion was measured by the slowing of the transit time up to total cerebral perfusion loss until the non-invasive Doppler ultrasound methods of perfusion measurement became available. Functional imaging, at first using PET, later fMRI, has added new dimensions to the investigation of brain function in coma.

Data derived from these newer methods during the terminal course of comatose syndromes (terminal coma; imminent brain death) implicate that imaging methods can be utilized to define criteria for treatment success or treatment failure which can be detected in an early stage (e.g., catastrophic brain lesions) and can be used for confirmation and documentation of clinical findings. The findings and their technical implementation are focused on better understanding of brain reactions to the primary disease process and its dynamics during treatment.

One of the goals of imaging methods is to give a basic view for the detection of loss of function, the documentation of irreversible loss of function as well as the irrefutable documentation of the time point of the total and irreversible loss of brain function which is needed in the determination of brain death to document the cause of death and to reliably assess the absence of therapeutic options.

Examinations pertaining to morphological finding of the spinal cord in the phase of spinalisation, i.e. after declaration of brain death have not been published so far. Neuropathological findings in brain death have been referred by Wijdicks and Pfeifer (Wijdicks and Pfeifer 2008).

#### CT-based imaging

Since the early 1980s, numerous investigations have been performed in this area (Rappaport et al. 1978). From the beginning on, the main interest lay in the perfusion of the large intracranial cerebral arteries as there was hope, that at that time used catheter angiographies could be avoided (Vatne et al. 1985).

The present situation demonstrates that the most valid criteria in CT and spiral CT are the diffuse swelling of the brain, and the narrowing of the ventricles (up to “brain tamponade” (Shiogai at al.) whereas the 2 phase CT demonstrates the loss of perfusion of the MCA and the perfusion loss of the large cerebral veins (Leclerc et al. 2006; Dupas et al. 1998). The value of CT angiography (CTA) has greatly improved (Combes et al. 2007; Leclerc 2007; Shemie et al. 2008; Welschehold et al. 2013). Studies testing CTA versus DSA versus TCD in patients who fulfill the criteria of brain death (clinically, by TCD, by EEG and DSA) and show cerebral perfusion stop at the level of the foramen magnum or the carotid siphon demanded loss of perfusion on CTA of the following arteries to prove cerebral perfusion stop: A2-ACA, M4-MCA, basilar artery, inner veins, and large brain veins and sinuses. There are, however, critical reports stating that, in the presence of isoelectric EEG, there are still up to 50% deviations from the goal of examination (Quesnel et al. 2007). However, in control investigations 2 to 24 hours later, total loss of cerebral perfusion can be documented. This indicates that there may be need for follow up of imaging in specific coma syndromes.

#### MRI-based imaging

MRI methods also document signs indicating the loss of cerebral function (Matsumura et al. 1996; Karantanas et al. 2002; Tollard et al. 2009; Nichol et al. 2011; Choi et al. 2010). Typical signs in the presence of severe brain swelling due to diffuse severe brain damage, such as in severe anoxic encephalopathy are: Swelling of the cortical and cerebellar gyri, loss of differentiation between grey and white substance, loss of intracranial contrast medium enhancement with demonstration of the “MRI hot nose sign” (referring to absent metabolic activity in the brain in the presence of preserved metabolic activity in the nose area), narrow ventricles, narrowing of basal (peripontine and mesencephalic) cisterns, diencephalic trantentorial herniation, and herniation of the cerebellar tonsils through the foramen magnum. The herniation of the cerebellar tonsils is considered to be the strongest sign in brain death humans. On MR angiography, the supraclinoidal arteries are not detectable; no signs of flow void of intracranial arteries are present (Karantanas et al. 2002; Ishii et al. 1996).

In patients suffering from severe TBI, multimodal MRT imaging (e.g., ^1^H-spectroscopy, diffusion tensor imaging) seems to increase the specificity of prognostication up to 97% (Tollard et al. 2009; Nichol et al. 2011). Criteria for the diagnosis of brain death are still pending.

#### HMPAO SPECT

Scintigraphic methods have been used since the 1980s. In the early phases, the method was employed only in cases in which total loss of cerebral function was suspected, and a DSA was not indicated. Improved technology including bedside methods has allowed for further progress of the method. A number of controlled trials have been performed in patients examined after the diagnosis of brain death was made. Scintigraphic tests were compared to DSA. The underlying diseases included subarachnoid hemorrhages, ICH, TBI, acute anoxic brain damage, brain tumors, and strokes (Kurtek et al. 2000). Reliable signs of cessation of cerebral perfusion are considered to be i) the “hot nose” sign and ii) the “empty skull” sign when hemispheres, brain stem and cerebellum are examined and no activity is detectable.

#### Xenon CT

Xenon CT can be utilized for confirmation of loss of cerebral function; however, the method is mainly recommended for use prior to the determination of brain death (Ashwal et al. 1989; Pistoia et al. 1991). The amount of metabolic activity measured by this method correlates with the diagnosis and prognosis of the condition: i) < 5 ml/min/100 g is consistent with no flow sign of brain death (for children and adults); ii) <10 ml/min/100 g is compatible with the complete and irreversible loss of brain function (for children); and iii) 10–15 ml/min/100 g is consistent with an improved prognosis.

#### Summary

The signs found in CT and multimodal MRI are complimented by the signs of an irreversible course which is documented by diffuse brain swelling, compression of the ventricles and basal cisterns as well as by the herniation of cerebellar tonsils through the foramen magnum.

CT angiography, MRI angiography, HMPAO SPECT and Xenon CT are methods suitable to document cessation of intracranial perfusion and can be used at least in questionable cases to augment or confirm the required diagnostic tests.

The recommendations of the Canadian Critical Care Society (Shemie et al. 2008) suggests a stepwise use of imaging methods in the neurological diagnosis of brain death, with specific use in the documentation of loss of cerebral perfusion: a.) Radionuclide angiography or CT angiography; b.) Four-vessel angiography; c.) MRI angiography or Xenon CT; c.) If a-c cannot be performed, cardiac arrest is considered equal proof (e.g., in the case of non-heart-beating donors).

#### Comment

One should be aware of the current discussion in the USA that has been published by the President’s Council on Bioethics in December 2008. It has been proposed that the diagnosis “total and irreversible brain failure” should be separated from the “neurological determination of death” (NDD), which is performed using this diagnosis. There is a new discussion on the problem of decision making on the treatment in patients suffering from a terminal coma or imminent brain death. Criteria of the FOUR score include a catastrophic brain lesion, which has to be documented by adequate imaging techniques, e.g., multimodal MRT imaging (Nichol et al. 2011). This line of thought would become relevant for the procedure of organ donation. It seems to be necessary to develop much more sophisticated knowledge on the preterminal signs of the various coma forms, i.e. clinical and imaging experience.

## Discussion

The compiled data of imaging methods in the context of coma is provided to give an overview of the current knowledge concerning the contribution of brain imaging methods in coma and related states. The focus of the article is not to provide an exhaustive review of all of the various facets of brain imaging in single disease entities, but rather to give information on the approaches to the differentiation of comatose states by imaging methods. Therefore, no attempt was made to describe the vast information gathered in specific disease entities such as stroke or brain tumors, much less in traumatic brain injury. The approach to the clinical sign “coma” is a clinically relevant task, which requires several different approaches. The first and most important approach is the clinical examination, which can differentiate between supratentorial, infratentorial and diffuse brain damage. With this information, it becomes possible to narrow down the differential diagnosis to certain etiologies and brain regions in which the primary cause of disease should be sought. The contribution of imaging methods to this differential diagnostic approach is compiled. The next step is the assessment of disease prognosis. This question can again be based on the clinical findings, and imaging methods can used in certain areas to enhance the clinically assessed prognostic data. Here, certain neurophysiological methods render the most relevant prognostic statements. Imaging methods are especially valuable in the prognostic assessment of PVS and MCS, an area where neurophysiological methods provide little information. The proper selection of examination methods for diagnostic and prognostic purposes in coma and related states requires broad knowledge of the strengths and weaknesses of the various methods. Since the various examination methods are performed by a number of different medical subspecialties, the neurologist caring for comatose patients is in need of knowledge as to what examinations to have performed and for which purpose. As in many circumstances in critical care medicine, the unreflected application of a host of possible diagnostic tests does lead to a better understanding of the disease process per se. The correct selection of technical examinations, depending on the nature of the clinical problem on hand, renders highly sensitive and reliable information in many specific clinical situations. This is especially true in the diagnosis of the terminal phase of comatose patients where multimodal MRI methods may increase the prognostic accuracy earlier in the clinical course.
